# Off-target effects of sodium-glucose co-transporter 2 blockers: empagliflozin does not inhibit Na^+^/H^+^ exchanger-1 or lower [Na^+^]_i_ in the heart

**DOI:** 10.1093/cvr/cvaa323

**Published:** 2020-11-02

**Authors:** Yu Jin Chung, Kyung Chan Park, Sergiy Tokar, Thomas R Eykyn, William Fuller, Davor Pavlovic, Pawel Swietach, Michael J Shattock

**Affiliations:** 1 British Heart Foundation Centre of Research Excellence, King’s College London, The Rayne Institute, St Thomas’ Hospital, Lambeth Palace Road, London SE1 7EH, UK; 2 Department of Physiology, Anatomy and Genetics, Parks Road, Oxford, OX1 3PT, UK; 3 School of Biomedical Engineering and Imaging Sciences, King’s College London, The Rayne Institute, St Thomas' Hospital, Lambeth Palace Road, London SE1 7EH, UK; 4 Institute of Cardiovascular & Medical Sciences, Sir James Black Building, University of Glasgow, University Avenue, Glasgow G12 8QQ, UK; 5 Institute for Cardiovascular Sciences, University of Birmingham, Wolfson Drive, Birmingham B15 2TT, UK

**Keywords:** Heart failure, SGLT2 inhibitor, Na/H exchanger-1, Intracellular Na, NMR spectroscopy

## Abstract

**Aims:**

Emipagliflozin (EMPA) is a potent inhibitor of the renal sodium-glucose co-transporter 2 (SGLT2) and an effective treatment for type-2 diabetes. In patients with diabetes and heart failure, EMPA has cardioprotective effects independent of improved glycaemic control, despite SGLT2 not being expressed in the heart. A number of non-canonical mechanisms have been proposed to explain these cardiac effects, most notably an inhibitory action on cardiac Na^+^/H^+^ exchanger 1 (NHE1), causing a reduction in intracellular [Na^+^] ([Na^+^]_i_). However, at resting intracellular pH (pH_i_), NHE1 activity is very low and its pharmacological inhibition is not expected to meaningfully alter steady-state [Na^+^]_i_. We re-evaluate this putative EMPA target by measuring cardiac NHE1 activity.

**Methods and results:**

The effect of EMPA on NHE1 activity was tested in isolated rat ventricular cardiomyocytes from measurements of pH_i_ recovery following an ammonium pre-pulse manoeuvre, using cSNARF1 fluorescence imaging. Whereas 10 µM cariporide produced near-complete inhibition, there was no evidence for NHE1 inhibition with EMPA treatment (1, 3, 10, or 30 µM). Intracellular acidification by acetate-superfusion evoked NHE1 activity and raised [Na^+^]_i_, reported by sodium binding benzofuran isophthalate (SBFI) fluorescence, but EMPA did not ablate this rise. EMPA (10 µM) also had no significant effect on the rate of cytoplasmic [Na^+^]_i_ rise upon superfusion of Na^+^-depleted cells with Na^+^-containing buffers. In Langendorff-perfused mouse, rat and guinea pig hearts, EMPA did not affect [Na^+^]_i_ at baseline nor pH_i_ recovery following acute acidosis, as measured by ^23^Na triple quantum filtered NMR and ^31^P NMR, respectively.

**Conclusions:**

Our findings indicate that cardiac NHE1 activity is not inhibited by EMPA (or other SGLT2i’s) and EMPA has no effect on [Na^+^]_i_ over a wide range of concentrations, including the therapeutic dose. Thus, the beneficial effects of SGLT2i’s in failing hearts should not be interpreted in terms of actions on myocardial NHE1 or intracellular [Na^+^].

## Graphical Abstract

**Figure:**
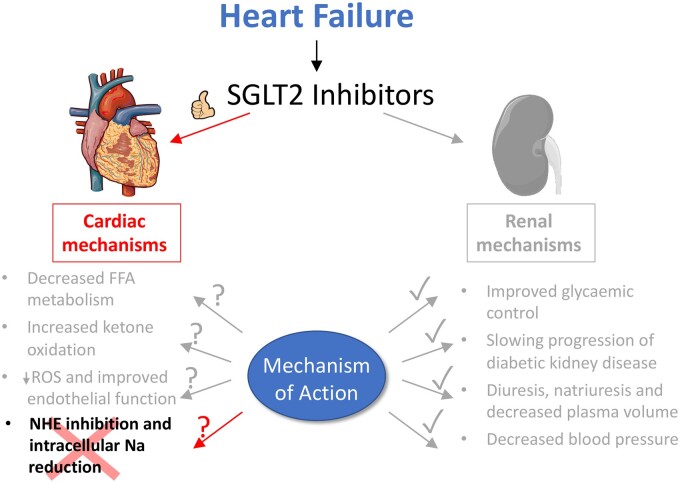

## 1. Introduction

Empagliflozin (EMPA), an inhibitor of renal sodium-glucose co-transporter 2 (SGLT2), is used clinically in the treatment of type-2 diabetes.^1^ Recent clinical trials have, however, described an unexpected beneficial cardiovascular effect associated with EMPA, notably a reduction in heart failure-related hospitalization independent of improved glycaemic control.^2^ These clinical observations are supported by preclinical studies in animal models, with or without background diabetes, showing that heart failure and pathological cardiac remodelling are alleviated by EMPA.^3–5^ These cardioprotective effects are observed despite cardiomyocytes not expressing SGLT2,^6–8^ leading to suggestions that SGLT2 inhibitors (SGLT2i’s) may have non-canonical actions on other proteins in the heart.

Using isolated cardiac ventricular myocytes, two previous studies reported a strong inhibitory class effect of SGLT2i’s (including EMPA) on cardiac Na^+^/H^+^ exchanger-1 (NHE1), a Na^+^-driven pH-regulating membrane protein.^9^^,^^10^ In these studies, SGLT2i’s were reported to reduce intracellular [Na^+^] and [Ca^2+^] as a result of NHE1 inhibition. Given that elevation of [Na^+^]_i_ is causally associated with the pathophysiology of heart failure,^11^^,^^12^ the authors suggested that the non-canonical efficacy of SGLT2i’s relates to their [Na^+^]_i_-lowering effect, leading to reduced Ca-overload and hence protection against adverse cardiac remodelling.^13^^,^^14^

There are, however, concerns about the feasibility of this hypothesis, based on the properties of NHE1. As the dominant isoform present in myocardium, NHE1 is steeply activated by a fall in intracellular pH (pH_i_).^15^ While intracellular acidosis activates NHE1 and drives a recovery of pH_i_ and intracellular Na^+^-loading, the activity of this transporter near resting pH_i_ (∼7.2) is very low (<1 mmol/min),^15^ and therefore inhibiting this basal flux would not be expected to meaningfully affect steady-state myocyte [Na^+^]_i_ while the Na^+^/K^+^ ATPase remains functional. In fact, numerous reports in the literature have shown that NHE1 inhibition in the normoxic myocardium with drugs, such as cariporide, is without effect on intracellular [Na^+^].^16–18^ The suggestion that SGLT2i’s reduce intracellular [Na^+^] via NHE1 inhibition under physiological conditions, in healthy normoxic myocytes or in a beating heart, is therefore surprising and merits further validation, particularly since this idea is gaining traction.^19–22^

In the present work, we evaluate the inhibitory effects of EMPA on cardiac NHE1 and further investigate its actions on cardiac Na^+^ handling in isolated cardiac myocytes and Langendorff-perfused hearts. Our findings show that EMPA, contrary to previously published reports, is not a direct inhibitor of cardiac NHE1 and does not reduce intracellular [Na^+^].

## 2. Methods

### 2.1 Approvals for animal work

Animal procedures were performed in compliance with the guidelines from Directive 2010/63/EU of the European Parliament on the protection of animals used for scientific purposes, Home Office Guidance on the Operation of the Animals (Scientific Procedures) Act of 1986 and the King’s College London and the University of Oxford institutional guidelines. Wistar rats, CD1 mice, and Dunkin-Hartley guinea pigs were purchased through Envigo. Sprague-Dawley rats were purchased through Charles River. Animals were killed humanely by a Schedule 1 method and death confirmed by exsanguination.

### 2.2 Drugs

EMPA was obtained independently from Boehringer Ingelheim^2^^,^^20^^,^^23^ and from MedChemExpress (MCE),^9^^,^^10^^,^^24^^,^^25^ and referred to herein as EMPA and mEMPA, respectively. Dapagliflozin (DAPA) and canagliflozin (CANA) were obtained from MCE. Cariporide, a selective NHE1 inhibitor, was obtained from Sanofi-Aventis. The structure and physical characteristics of EMPA were verified using high-pressure liquid chromatography-mass spectrometry (HPLC-MS), 1D/2D NMR and circular dichroism (CD) spectroscopy. Therapeutically relevant plasma concentrations of EMPA have been reported to be in the range ∼200–700 nmol/L with plasma protein binding of ∼90%.^26^ To ensure we adequately cover or exceeded both the concentrations used in previously studies *in vitro* and the therapeutically relevant concentrations, we have used concentrations ranging from 1 to 30 µM in DMSO (0.01% v/v, or 0.03% v/v in the case of 30 µM EMPA).

### 2.3 Cardiomyocyte isolation

Male Sprague-Dawley (300–325 g) rats, used for fluorescence experiments, were sacrificed by a method designated Schedule 1 by the Animals (Scientific Procedures) Act of 1986 (concussion of the brain by striking the cranium followed by cervical dislocation). Male Wistar (325–350 g) rats, used for electrophysiology experiments, were terminally anaesthetized and heparinized with 60 mg/kg sodium pentobarbitone and 100 U sodium heparin, intraperitoneally. Ventricular myocytes were isolated from Langendorff-perfused hearts using a combination of mechanical and enzymatic (1 mg/mL Type II Collagenase, Worthington Biochemical Corporation; 0.025 mg/mL Type XIV Protease, Sigma-Aldrich/Merck) dispersion. For cells isolated for fluorescence experiments, the isolation solution contained (in mM): 120 NaCl, 4 KCl, 1.2 MgCl_2_, 10 HEPES, 1 NaH_2_PO_4_, 11 glucose, 20 taurine, 2.5 pyruvic acid, (pH 7.4 with NaOH at 37°C). For cells isolated for electrophysiology, the isolation solution contained (in mM): 130 NaCl, 4.5 MgCl_2_, 4.2 HEPES, 0.4 NaH_2_PO_4_, 10 glucose, 20 taurine, 10 creatine (pH 7.4 with NaOH at 37°C) and bubbled with 100% O_2_. Isolated myocytes were introduced to calcium-containing solution in a step-wise manner to reach a final concentration of 1 mM. Only quiescent, Ca^2+^-tolerant myocytes that were rod-shaped with clear striations, were selected for experiments.

### 2.4 Cell culture

HCT116 cells (kind gift from Professor Walter Bodmer, University of Oxford, UK) were cultured in Dulbecco’s Modified Eagle’s Medium (DMEM) containing 44 mM bicarbonate, 10% foetal calf serum, and 1% pen-strep and maintained at 37°C and 5% CO_2_. Stably transfected HEK293-hSGLT2 cells were obtained from Boehringer Ingelheim. Cells were grown and maintained in DMEM at 37°C and 5% CO_2_.

### 2.5 Fluorescence imaging

For pH measurements, primary cardiomyocytes or HCT116 cells were loaded with 17.6 µM cSNARF-1 (AM-ester) at room temperature for 6 min, plated onto a poly-L-lysine coated Perspex chamber mounted on a Zeiss LSM 600 confocal system. Excitation was delivered by 555 nm laser and emission was collected simultaneously at 580 and 640 nm. For intracellular sodium ion concentration ([Na^+^]_i_) measurements, primary cardiomyocytes were loaded with 17.7 µM SBFI (AM-ester with 0.1% Pluronic) at room temperature for 2 h, plated onto a poly-L-lysine coated Perspex chamber mounted on an Olympus IX73 microscope. Excitation alternated between 365 nm for 100 ms and 385 nm for 200 ms, emission was collected at >500 nm.

### 2.6 Superfusion

All superfusion experiments were performed at 37°C achieved through a feedback heater. Dye-loaded cells were superfused in Tyrode’s buffer containing (in mM): 135 NaCl, 4.5 KCl, 1 MgCl_2_, 20 HEPES, 11 glucose, and 1 CaCl_2_ (pH 7.4 with NaOH at 37°C; and, in the case of SBFI, 2 mM probenecid dissolved in NaOH). For the ammonium pre-pulse protocol, cells were stabilized in Tyrode’s buffer for 5 min, perfused in 15 mM ${\text{NH}}_{4}^{+}$Cl (NaCl reduced to maintain osmolarity) for 6 min, followed by return to Tyrode’s buffer (the recovery period). Washout of ${\text{NH}}_{4}^{+}$ produces a rapid rebound acidification which triggers NHE1 activity. Drugs were included in both NH_4_Cl-containing buffer and during the recovery period. For the acetate protocol, cells were stabilized in Tyrode’s buffer for 5 min, pre-treated in drug (or DMSO) for 10 min, then perfused in 80 mM acetate for 4 min (NaCl reduced to maintain osmolarity). For 0Na0Ca protocol, cells were stabilized in Ca-free Tyrode’s buffer for 4 min, superfused in Na-free/Ca-free buffer (in mM: 140 N-methyl-D-glucamine, 4.5 KCl, 1 MgCl, 20 HEPES, 11 glucose, and 0.5 EGTA; pH 7.4 with HCl at 37°C) for 8 min, then returned to Ca-free buffer containing 30 µM cariporide for 8 min. Drugs were included throughout the protocol.

### 2.7 Electrophysiology

Na^+^/K^+^ pump current was measured using whole-cell voltage clamping, using the perforated patch technique to minimize intracellular dialysis and at 35°C.^24^ Rat ventricular cardiac myocytes were plated onto laminin (L2020 Sigma-Aldrich) coated coverslips and Na^+^/K^+^ pump current measured via borosilicate glass microelectrodes (1.5–2 MΩ). The pipette filling solution contained (in mM): 103 Cs methanesulfonate, 25 Na methanesulfonate, 20 CsCl, 10 HEPES, 1 CaCl_2_, 1 MgCl_2_ (pH = 7.3 with CsOH at 35°C). Before the experiment, amphotericin B (A4888 Sigma-Aldrich) was freshly dissolved in DMSO and added to the pipette solution (final concentration of ∼240 µg/mL). Whole-cell currents were monitored using an Axopatch 200B amplifier, Digidata 1322A DAQ and pClamp 9.2 software (Molecular Devices, Sunnyvale, CA). Bath and pipette solutions were designed to minimize contamination with other currents. The bath solution contained (in mM): 140 NaCl, 5 KCl, 1 MgCl_2_, 2 NiCl_2_, 1 BaCl_2_, 10 glucose, 10 HEPES (pH 7.3 with NaOH at 35°C). In the majority of experiments, the membrane potential was clamped at 0 mV. Current–voltage (I/V) relationships were recorded during a 10 s ramp pulse (13 mV/s) from +40 to −90 mV. Na^+^/K^+^ pump current was calculated after subtracting the current recorded in external K^+^-free solution from that recorded in the presence of 5 mM of K^+^. Na^+^/K^+^ pump current at 0 mV was low-pass filtered at 100 Hz and sampled at 200 Hz. During ramp I/V curves, currents were low-pass filtered at 1 kHz and sampled at 2 kHz. In order to average between cells, and provide mean ± error bars, data were ‘binned’ along the X-axis into ‘bins’ each containing 800 samples with each bin representing an average of 5.2 mV.

### 2.8 Glucose uptake assay

One day prior to experiments, cells overexpressing the human SGLT2 transporter (HEK293-hGSLT2) were seeded to 75% confluency in a 96-well plate. Cells were washed in 1× phosphate buffered saline and glucose starved for 30 min in glucose-free DMEM and subsequently treated with either 5 or 50 nM EMPA, DAPA, or CANA for 15 min. Glucose uptake assay was performed using the Promega Glucose Uptake-Glo Assay following manufacturer’s instructions. The assay measures luciferase bioluminenscence following uptake of 2-deoxyglucose into cells. Luminescence was recorded using the Promega GloMax Multi Detection System microplate reader.

### 2.9 Langendorff perfusion

Hearts were rapidly excised and cannulated via the aorta and perfused at 37°C at constant pressure of 80 mmHg (60 mmHg for guinea pig). Perfusion buffer was either Krebs–Henseleit buffer (KHB) containing (in mM): 118 NaCl, 5.9 KCl, 1.16 MgSO_4_, 25 NaHCO_3_, 0.48 EDTA, 11 glucose, 2.2 CaCl_2_, and bubbled with 5% CO_2_/95% O_2_ or modified Tyrode’s buffer containing (in mM): 135 NaCl, 4.5 KCl, 1.16 MgCl_2_, 10 HEPES, 0.48 EDTA, 11 glucose, 2.2 CaCl_2_ (pH 7.4 with NaOH at 37°C), and bubbled with 100% O_2_. For the Na-acetate protocol, Tyrode’s buffer was modified to 10 mM Na-acetate and 125 mM NaCl. Hearts were subject to one of two protocols. Protocol 1: 20 min stabilization in KHB then 15 min KHB with 1 µM EMPA, DMSO (0.01% v/v), or 10 µM cariporide. Protocol 2: 10 min stabilization in KHB, 10 min stabilization in Tyrode’s buffer, 15 min in Tyrode’s buffer containing 1 or 10 µM EMPA, DMSO or 10 µM cariporide, then 40 min in 10 mM Na-acetate buffer supplemented with drug. Cardiac function was monitored throughout the protocol via a balloon inserted into the left ventricle and monitored on LabChart. The Langendorff rig was modified for NMR as described previously.^27^

### 2.10 NMR spectroscopy

^31^P and ^23^Na NMR spectra were acquired on the Bruker Avance III 400 MHz Spectrometer 9.4 T vertical-bore magnet. For mouse experiments, 10 mm dual tuned ^31^P/^1^H and 15 mm single tuned ^23^Na coils were used. For rat, 15 mm dual tuned ^31^P/^1^H and ^23^Na/^1^H coils were used. For guinea pig, a 30 mm dual tuned ^31^P/^23^Na microimaging coil was used. Spectra were acquired and analysed using TopSpin 3.7pl software. For every heart, shimming was carried out on either the ^1^H channel or the ^23^Na channel. Time-resolved ^23^Na spectra were acquired using multiple quantum filtered experiments to separate intracellular and extracellular contribution to the ^23^Na signals, as described previously.^27^ Interleaved triple quantum filtered (TQF) and double quantum filtered NMR acquisitions, consisting of 192 scans and an experimental duration of 1 min (252 scans and 2 min for mouse), were recorded throughout the entire protocol. ^31^P spectra were acquired with a 60° flip angle, 64 scans, and a total experiment duration of 5 min for a single spectrum.

### 2.11 Statistics

Data are reported as mean ± SEM. For cardiomyocyte-based experiments, statistical testing was performed on number of cells. Control (DMSO) recordings were performed on each day to match data for the effects of drug. Hierarchical analysis was performed to seek evidence for clustering. Non-hierarchical tests were deemed suitable for datasets with small clustering (<15%).^28^ To compare between pH-flux relationships and between time courses, a two-way ANOVA followed by Sidak’s multiple comparison’s test was used to determine statistical significance. To compare steady-state pH, a one-way ANOVA followed by Tukey’s multiple comparison’s test was used to determine statistical significance. To compare pH_i_ recovery time course, left ventricular developed pressure (LVDP) and coronary flow in Langendorff perfused hearts, a two-way ANOVA followed by Tukey’s multiple comparison’s test was used to determine statistical significance. For all other assays, two-tail, unpaired student’s *t*-test was used to compare SGLT2i group mean with the control (DMSO) group. *P <* *0.05, **P <* *0.01, ***P <* *0.001, ****P <* *0.0001. Data analysis was carried out in a blinded manner. For experiments on single cells, repeats are noted as *n* = number of animals or *n* = number of cells from number of animals from which cells were isolated.

## 3. Results

### 3.1 EMPA does not inhibit NHE1 in isolated cardiomyocytes

EMPA, sourced from two independent manufacturers (mEMPA, EMPA), was first tested for purity by 1D ^1^H and ^13^C NMR acquired at 800 MHz, CD and HPLC-MS (Supplementary material online, *Figure S1*). These assays showed that both sources supplied the same chemical compound (Supplementary material online, *Figure S1A, C,* and *D*) in a single enantiomeric form (Supplementary material online, *Figure S1B*), with impurities <1% determined by NMR (w/w). Both EMPA and mEMPA inhibited glucose uptake in a dose-dependent manner in HEK293 cells overexpressing SGLT2, thus verifying that the drugs were active compounds (Supplementary material online, *Figure S2*).

**Figure 1:**
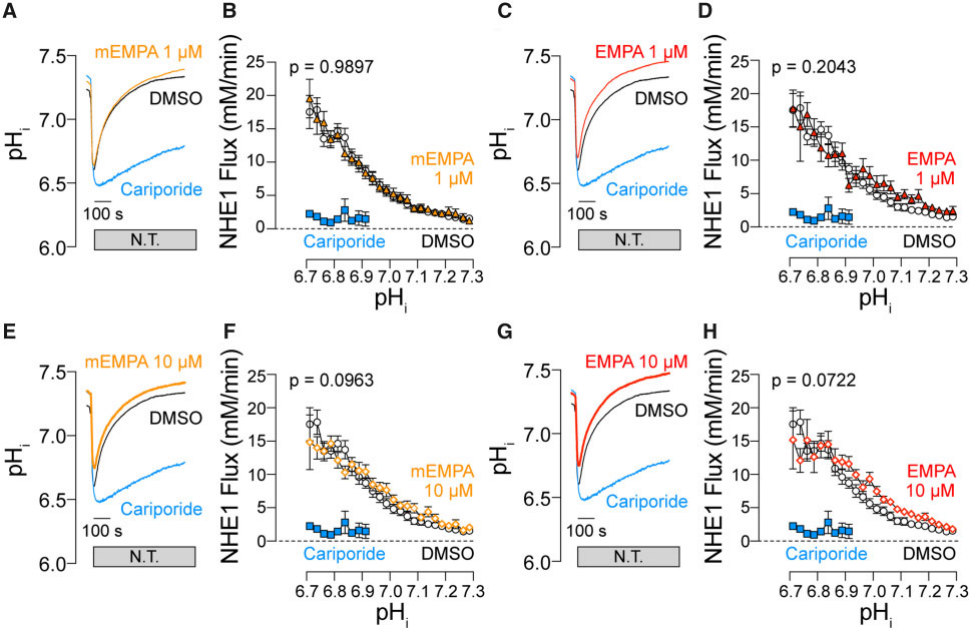
EMPA does not inhibit NHE1 activity in isolated cardiomyocytes. pH_i_ recovery time course (*A*), (*C*), (*E*), (*G*) and NHE1 flux as a function of pH_i_ (*B*), (*D*), (*F*), (*H*) measured in rat ventricular cardiac myocytes exposed to acute NH4^+^ acid-load and wash out and pre-treated with DMSO, 10 µM cariporide or 1 or 10 µM EMPA obtained from MedChemExpress (mEMPA; yellow) or Boehringer (EMPA; red). Flux is calculated from rate of pH_i_ change (from time courses) multiplied by buffering capacity (obtained in previous studies). Note: in the presence of cariporide, pH_i_ recovery is not complete in 12 min, and the flux is therefore only plotted for pH < 6.9. N.T., Normal Tyrode’s buffer. All data means ± SEM [error bar not included in *(A), (C), (E)*, and *(G)* for clarity]. *n* = 14–20 cells from three rats per condition.

**Figure 2:**
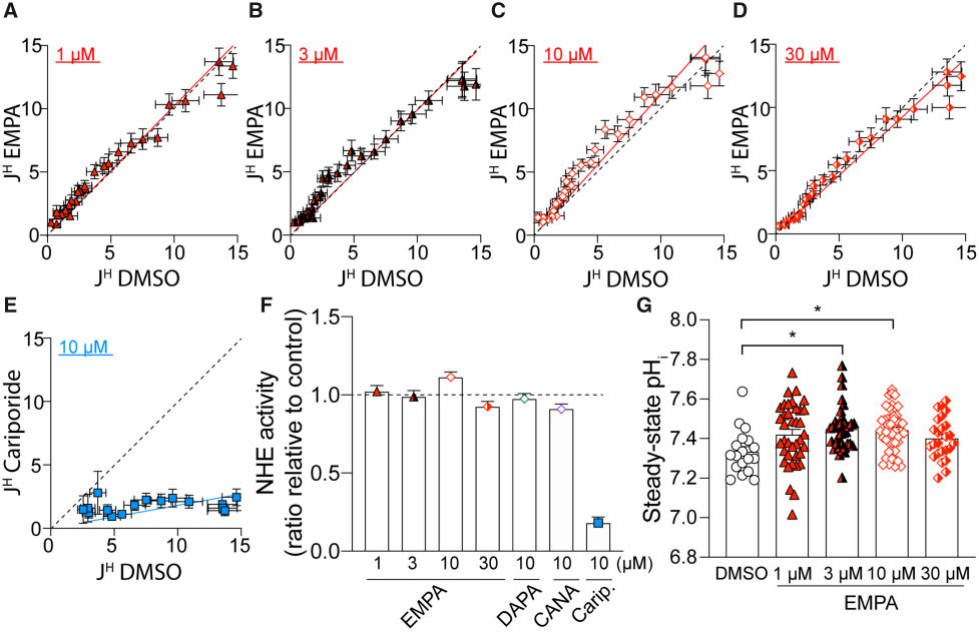
EMPA does not inhibit NHE1 flux in isolated cardiomyocytes. (*A–E*) Acid extrusion flux (J^H^; mM/min) plotted at matching pH_i_ for control (DMSO) and in presence of drug (EMPA or cariporide) at the concentrations indicated. Red or blue lines show best fit through origin, and the slope describes NHE1 activity in the presence of drug (where 1.0 is no inhibition, indicated in broken black line). (*F*) Slope of flux–flux relationships for EMPA, DAPA, CANA, and cariporide (Carip.). (*G*) Steady-state pH_i_ attained at the end of pH recovery. All data are means ± SEM; means ± 95% confidence interval for (*F*). Data from EMPA and mEMPA were combined. For EMPA and cariporide, *n* = 30–49 cells from three rats per condition. For DAPA and CANA, *n* = 31–43 cells from two rats per condition. *P <* *0.05 by one-way ANOVA.

Having validated the chemical identity and purity of EMPA, the effects of EMPA on NHE1 activity was investigated in isolated cardiac myocytes using fluorescence measurements on cells loaded with the pH-reporter cSNARF1 (*Figure 1*). The inhibitory effects of compounds on NHE1 are resolved best under conditions that normally evoke large NHE1 fluxes, such as in response to an intracellular acidosis. Cells were acid-loaded by means of an ammonium pre-pulse solution manoeuvre. Upon ammonium washout, the cell abruptly acidifies as intracellular ${\text{NH}}_{4}^{+}$ deprotonates to membrane-permeable NH_3_ which then rapidly escapes across the membrane leaving H^+^ ions in the cytoplasm (the acid-load). To inactivate ${\text{HCO}}_{3}^{-}$−dependent acid-extruders, myocytes were superfused in CO_2_/bicarbonate-free HEPES-buffered Tyrode’s buffer. The time course of pH_i_ recovery provides a readout of NHE1 activity. In control cells, pH_i_ recovered towards pH_i_ 7.3 over a period of ∼12 min. Cariporide (10 µM), a potent and selective NHE1 inhibitor,^29^ substantially inhibited pH_i_ recovery, explaining why pH_i_ remained acidic during the measurement period (*Figure 1A, C, E, and G*). Superfusion with buffers containing EMPA (1 or 10 µM) did not slow the rate of pH_i_ recovery. From these recovery time courses, transmembrane H^+^ flux was calculated and plotted as a function of pH_i_ (*Figure 1B, D, F, and H*). Briefly, flux is obtained by multiplying the rate of pH_i_ recovery (dpH_i_/dt) and buffering capacity (dH/dpH) for rat myocytes determined in an earlier study.^30^ NHE1-mediated transmembrane H^+^ flux was unaffected by either mEMPA or EMPA, strongly arguing against any direct inhibitory actions on cardiac NHE1. This is visualized by plotting flux in the presence of EMPA vs. flux at matching pH_i_ recorded under drug-free conditions (*Figure 2A–E*); the slope of this relationship, fitted from the origin, gives an estimate of NHE1 activity in the presence of drug across all pH_i_ values tested. Given that neither EMPA nor mEMPA appeared to have an effect on NHE1 flux, data from both drugs at their respective concentrations were combined. For 1, 3, and 10 µM EMPA, the 95% confidence interval of the slope of the flux–flux relationship included 1.0, i.e. no significant inhibition by EMPA (*Figure 2F*). At supra-therapeutical concentration of 30 µM EMPA (*Figure 2D*), the 95% confidence interval was 0.89–0.96, i.e. a statistically significant inhibition, but the magnitude of this inhibitory effect was only 8% [compared to effects of cariporide (*Figure 2E*) where 10 µM inhibited flux by >80%]. Interestingly, the steady-state pH_i_ reached at the end of pH_i_ recovery was significantly more alkaline, by 0.1 units, in 3 and 10 µM EMPA (*Figure 2G*). Whilst this observation is not consistent with NHE1 inhibition, a possible explanation for the small change in steady-state pH_i_ is an inhibitory effect of EMPA on metabolic acid production or acid-loading transporters, such as Cl^−^/OH^−^ exchange.

### 3.2 Other SGLT2 inhibitors do not affect NHE1 flux

Uthman *et al.*^10^ reported that two other SGLT2i’s, DAPA and CANA, also inhibit NHE1. Given that in our hands EMPA did not affect NHE1 activity, we tested whether DAPA or CANA inhibit NHE1 in cardiac myocytes subjected to intracellular acidification via an ammonium pre-pulse manoeuvre. As seen with EMPA, neither of these SGLTi’s (10 µM) inhibited pH_i_ recovery nor meaningfully reduce NHE1 flux (Supplementary material online, *Figure S3*).

### 3.3 EMPA does not block flux carried by the human NHE1 protein

Although EMPA did not inhibit NHE1 flux in isolated rat myocytes, subtle species-specific differences in NHE1 primary structure may affect the protein-EMPA interaction. To exclude a species-specific interaction, NHE1 activity was measured in HCT116 cells, which express the human isoform of NHE1, in the presence or absence of EMPA. Neither 1 nor 10 µM EMPA inhibited pH_i_ recovery or NHE1 flux following intracellular acidification (Supplementary material online, *Figure S4*), suggesting that EMPA also does not inhibit the human isoform of NHE1.

### 3.4 EMPA does not affect cellular Na-handling in cardiomyocytes

**Figure 3:**
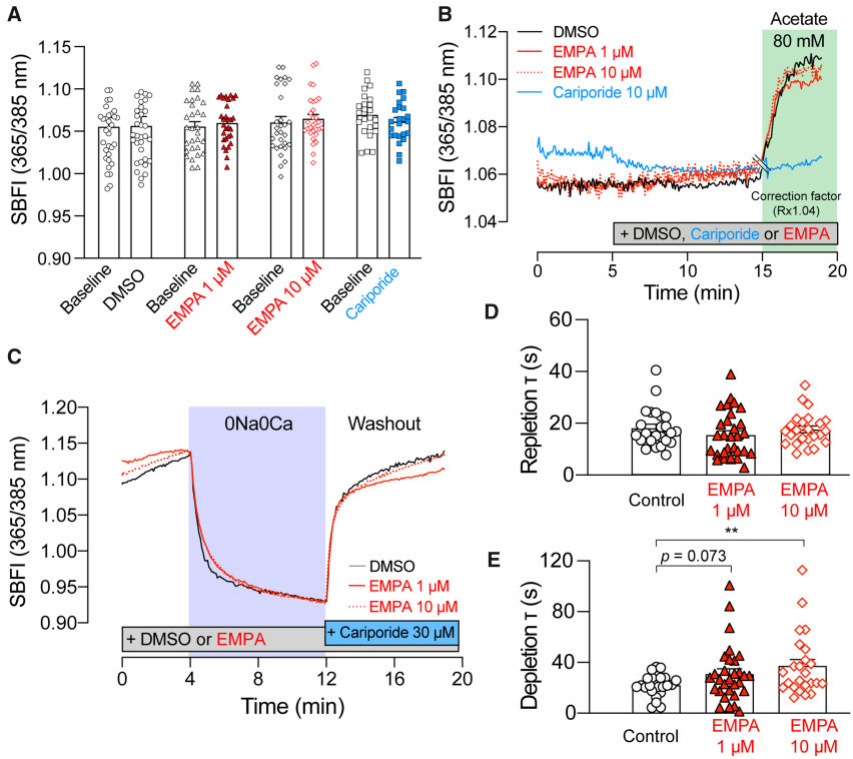
EMPA does not affect intracellular Na^+^ handling in isolated cardiomyocytes. Intracellular [Na^+^] measured using SBFI fluorescence in myocytes treated with DMSO, 1 or 10 µM EMPA or 10 µM cariporide (*A*) at baseline or (*B*) in 80 mM Na-acetate. Ratio corrected for pH-artefact (Rx1.04), marked by double line at 15 min. See Supplementary material online, *Figure S2* for original time course. *n* = 24–33 cells from three rats per condition. (*C*) Interrogation of NHE1-independent Na^+^ transport pathways using 0Na0Ca protocol. Time constant of (*D*) Na^+^ re-uptake and (*E*) Na^+^ efflux. All data are means ± SEM [error bar not included in (*B*) and (*C*) for clarity]. *n* = 24–30 cells from three rats per condition. **P <* *0.01 by two-tail, unpaired student’s *t*-test.

Another readout of NHE1 activity is [Na^+^]_i_ measured by SBFI. Baseline [Na^+^]_i_ was recorded and found to be unaffected by EMPA at neither 1 nor 10 µM (after 15 min pre-treatment in EMPA; *Figure 3A*). Similarly, 10 µM cariporide for 15 min had no significant effect on baseline [Na^+^]_i_. To drive an NHE1-dependent rise in [Na^+^]_i_, myocytes were superfused with 80 mM acetate in HEPES-buffered solution, which acidifies the cytoplasm and triggers NHE1 activity and subsequent intracellular sodium loading (*Figure 3B*). The acetate-evoked [Na^+^]_i_ response was ablated by cariporide, revealing an acidification-induced pH-artefact of the dye (Supplementary material online, *Figure S5*). The presence of EMPA did not affect the slope of the [Na^+^]_i_ rise compared to the control, consistent with results obtained with NHE1 interrogated using pH_i_ as a readout of activity in *Figure 1*. The time courses presented in *Figure 3B* have been corrected for this pH-dependence of SBFI ratio.

**Figure 4:**
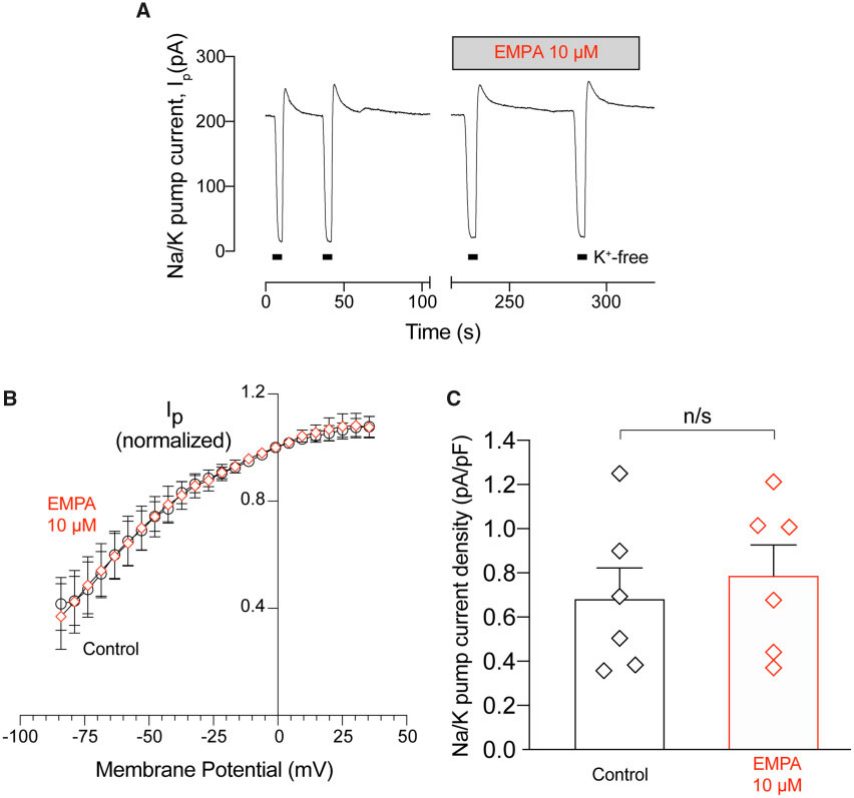
EMPA does not affect Na^+^/K^+^ ATPase current in isolated cardiomyocytes. Whole-cell pump current was measured using the perforated patch technique in rat ventricular myocytes. (*A*) Representative trace showing pump current (I_p_) recorded under control conditions and after the application of EMPA (10 µM). Pump current was recorded at 0 mV. Potassium-free bath solution was applied at the points shown (K^+^-free). (*B*) Current–voltage relationships recorded during the application of a negative-going voltage ramp (13 mV/s) under control conditions and after application of EMPA. Data were averaged (factor 800) to obtain ∼1 reading every 5 mV and normalized to the current reading at 0 mV. (*C*) Average Na^+^/K^+^ ATPase current density recorded after 300 s in control and EMPA. All data are means ± SEM. *n* = 6 cells from six rats.

NHE1 is one of a number of Na^+^-entry pathways into the myocyte, and it is plausible that EMPA may be affecting other transporters.^31^ To test this, myocytes loaded with SBFI were first perfused in Ca^2+^-free Tyrode’s buffer, then switched to buffer in which Na^+^ salts were replaced with N-methyl-D-glucosamine (Na^+^-free, Ca^2+^-free Tyrode’s buffer, 0Na0Ca; *Figure 3C*). This manoeuvre depleted intracellular Na^+^, and the rate of this depletion is an approximate readout of Na^+^/K^+^ ATPase (NKA) activity. Next, cells were returned to Na^+^-containing superfusates supplemented with 30 µM cariporide to allow Na^+^ entry by all routes, except for NHE1. EMPA (1, 10 µM) did not affect the time constant of Na^+^ repletion (*Figure 3D*), indicating that other Na entry routes are unlikely to be targets of EMPA. However, the rate of Na^+^-depletion was reduced by 10 µM EMPA (*Figure 3E*), suggesting a possible inhibition of NKA. Therefore, the activity of NKA was further probed using the perforated-patch voltage-clamp technique in rat cardiac myocytes to measure NKA current (*Figure 4*). Pump current was not affected by 10 µM EMPA treatment over the physiological voltage range (*Figure 4A and B*).

NKA current also provides another indirect index of [Na^+^]_i_. In these experiments, the perforated-patch method of voltage clamping was used to prevent cell dialysis.^32^ Under these conditions, NKA current is steeply dependent on the prevailing [Na^+^]_i_.^33^*Figure 4C* shows absolute pump current density measured in pA/pF. An EMPA-induced decline in [Na^+^]_i_ would be expected to reduce NKA current density at 0 mV; however, this was not observed, suggesting that EMPA did not lower [Na^+^]_i_ with respect to baseline in these experiments.

### 3.5 EMPA does not decrease intracellular [Na^+^] in Langendorff-perfused hearts

**Figure 5:**
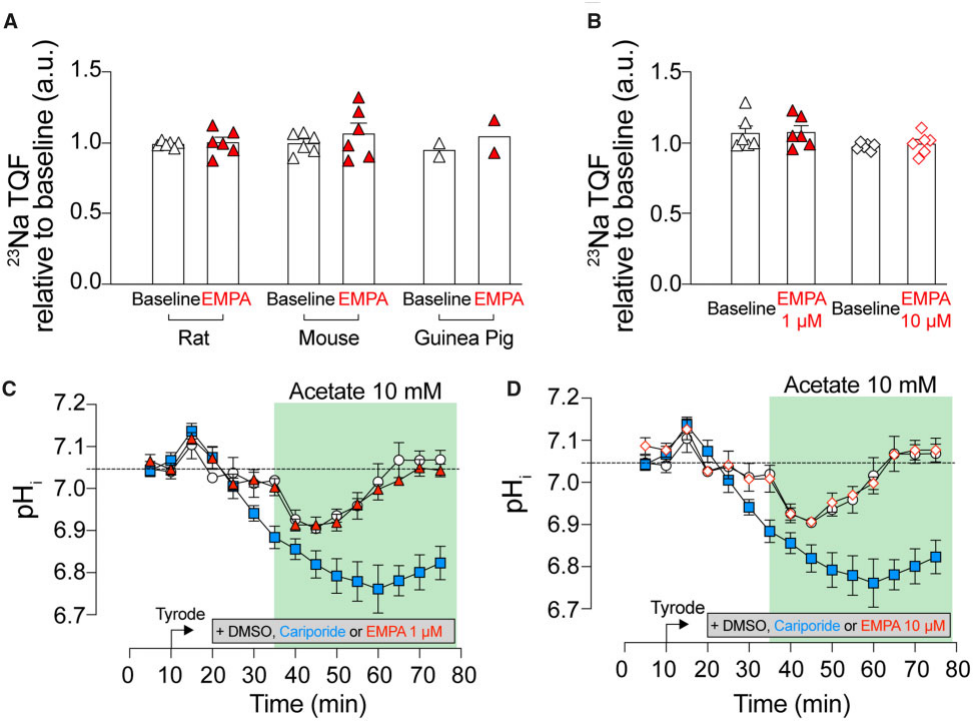
EMPA does not affect intracellular [Na^+^] and pH_i_ in Langendorff-perfused hearts. Intracellular [Na^+^] measurement using ^23^Na NMR spectroscopy in (*A*) rat, mouse and guinea pig hearts perfused in CO_2_/bicarbonate-buffered KHB and treated with 1 µM EMPA and (*B*) rat hearts perfused with CO_2_/bicarbonate-free Tyrode’s buffer and treated with either 1 or 10 µM EMPA. *n* = 6 rats (*A* and *B*), 6 mice (*A* and *B*), and 2 guinea pigs (*A*) per group; ^23^Na signal normalized to baseline. Rat hearts pre-treated with DMSO (open circle), 10 µM cariporide (blue square), (*C*) 1 µM or (red triangle) (*D*) 10 µM EMPA (red, open diamond), and perfused in Tyrode’s buffer supplemented with 10 mM Na-acetate. Intracellular pH measured using ^31^P NMR spectroscopy. All data mean ± SEM. *n* = 6 rats per group per assay.

Although EMPA had no effect on basal [Na^+^] in quiescent cardiomyocytes (*Figure 2A*) and two independent readouts of NHE1 activity in isolated myocytes (*Figures 1–3B*), some actions may become apparent only in the intact beating heart. Langendorff-perfused rat hearts were treated with EMPA, and [Na^+^]_i_ was measured using TQF NMR experiments.^27^ Following 20 min of stabilization in either CO_2_/bicarbonate-buffered Krebs (KHB) buffer or 10 min in CO_2_/bicarbonate-free HEPES-buffered Tyrode’s buffer, hearts were treated with either EMPA or DMSO for 15 min. Intracellular [Na^+^] remained unchanged during this period in hearts treated with either 1 or 10 µM EMPA (*Figure 5A and B*). EMPA also had no effect on [Na^+^]_i_ in mouse and guinea pig hearts, excluding a possible species-dependent response to EMPA (*Figure 5A*). Together, these results suggest that EMPA does not affect [Na^+^]_i_ handling in the isolated beating heart under physiological heart rates (mouse paced at 550 b.p.m.; rat 350–400 b.p.m.; guinea pig 250–300 b.p.m.) and at 37°C.

The effect of EMPA on beating hearts was further investigated in terms of the time course of pH_i_ recovery following intracellular acidosis imposed by perfusion in 10 mM acetate, to activate NHE1 (*Figure 5C and D*). Using serially acquired ^31^P NMR scans, intracellular pH was calculated from the chemical shift difference between the phosphocreatine (PCr) and inorganic phosphate (P_i_) peak according to the relationship determined previously.^34^ To probe for pH_i_ changes arising solely from NHE1, hearts were Langendorff perfused in CO_2_/bicarbonate-free HEPES-buffered Tyrode’s buffer. During 40 min of treatment in acetate, the NHE1 inhibitor cariporide attenuated pH_i_ recovery following intracellular acidification (*Figure 5C and D*). However, EMPA did not inhibit this pH_i_ recovery (1 and 10 µM), consistent with the lack of inhibitory actions of EMPA on cardiac NHE1.

### 3.6 EMPA does not affect cardiac function and energetics in Langendorff-perfused hearts

**Figure 6:**
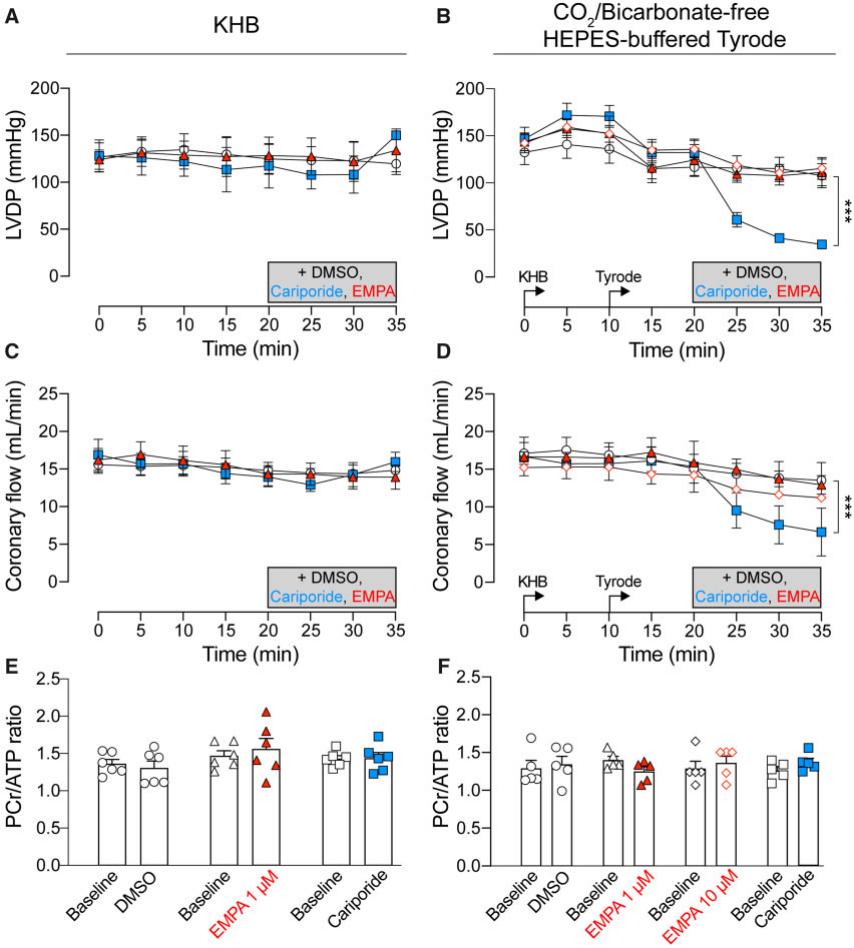
EMPA does not affect cardiac contractile function and energetics in Langendorff-perfused rat hearts. (*A, B*) LVDP measured using balloon inserted into left ventricle of rat heart. (*C, D*) Coronary flow required to maintain perfusion pressure at 80 mmHg. DMSO control (open circle), 10 µM cariporide (blue square), 1 µM EMPA (red triangle), or 10 µM EMPA (red, open diamond). (*E, F*) PCr/ATP ratio measured using ^31^P NMR spectroscopy. Hearts perfused in Kreb’s buffer (KHB) (*A*), (*C*), and (*E*) and in CO_2_/bicarbonate-free HEPES-buffered Tyrode’s (Tyrode) (*B*), (*D*), and (*F*). All data mean ± SEM. n = 6 rats (KHB) or 5 rats (CO_2_/bicarbonate-free Tyrode) per group. ***P <* *0.001 by two-way ANOVA.

LVDP was monitored by a balloon inserted into the left ventricle of Langendorff perfused rat hearts. Administration of EMPA did not affect LVDP (*Figure 6A and B*). The perfusion rate (i.e. a measure of coronary flow) that is required to maintain pressure at 80 mmHg was also not affected by EMPA treatment (*Figure 6C and D*), contrary to previous reports of a vasodilatory effect of SGLT2i’s.^10^ In contrast, cariporide reduced LVDP and coronary flow in hearts perfused with CO_2_/bicarbonate-free Tyrode’s buffer (*Figure 6B and D*). Cardiac energetics, measured in terms of the PCr/ATP ratio obtained through ^31^P NMR spectroscopy, was not affected by neither EMPA nor cariporide treatment (*Figure 6E and F*).

## 4. Discussion

In this study, we present results showing that EMPA, obtained from two independent sources, does not inhibit NHE1 measured in terms of the two readouts of its activity, pH_i_ and [Na^+^]_i_, in isolated cardiomyocytes or Langendorff-perfused beating hearts. We find that recovery of pH_i_ from an intracellular acid-load was not slowed to any meaningful degree in the presence of EMPA up to 30 µM (*Figures 1 and 2*). In addition to EMPA, two other SGLT2i’s, DAPA and CANA, were also tested for their proposed inhibitory actions on NHE1 activity and found that these, like EMPA, did not inhibit NHE1 flux (Supplementary material online, *Figure S3*). As a positive control of inhibition, cariporide (10 µM) substantially blocked pH_i_ recovery (*Figures 1 and 2E*). Intriguingly, steady-state pH_i_ attained at the end of the recovery period was modestly more alkaline in 3 or 10 µM EMPA, compared to drug-free conditions (*Figure 2G*). This effect cannot be explained by inhibitory actions on NHE1; rather, it may relate to an inhibitory effect on sarcolemmal acid-loaders or changes in intracellular proton production secondary to changes in metabolism.

In hearts Langendorff-perfused with CO_2_/bicarbonate-free Tyrode’s buffer, inhibition of NHE1 with cariporide (10 µM) resulted in intracellular acidification (*Figure 5C*) and a reduction in LVDP (*Figure 6D*), consistent with the effects of acidosis on cardiac contractility.^17^^,^^35^^,^^36^ Interestingly, this effect on contractility was not evident in cariporide-treated hearts perfused in KHB (compare *Figure 6C* vs. *D*), probably because the pH-regulating Na^+^/${\text{HCO}}_{3}^{-}$ co-transporter becomes activated in under these conditions and compensates for the loss of NHE1 activity. As the relationship between intracellular Na and contractility is steep,^37^ a ∼25% fall in intracellular [Na^+^], as previously reported,^9^^,^^10^ might be expected to be significantly negatively inotropic. However, we observed no effect of EMPA on LVDP (*Figure 6A and B*) in Langendorff-perfused hearts. Also, when measured directly, [Na^+^]_i_ was unaffected by EMPA at resting pH_i_ in isolated cardiomyocytes (*Figure 3A*) or in Langendorff-perfused hearts (*Figure 5C and D*). We also found no evidence that EMPA affects other Na^+^ entry pathways into the myocyte (*Figure 3D*). A potential effect of EMPA on Na^+^ extrusion via NKA observed in the SBFI loaded cardiomyocytes (*Figure 3E*) was not replicated in patch clamp experiments directly measuring NKA activity (*Figure 4*). The direct measurement of absolute NKA pump current (*Figure 4C*) also provides additional confirmation that EMPA did not lower [Na^+^]_i_ as this current is steeply dependent on the intracellular Na^+^ concentration.^33^

Prior to our study, EMPA was postulated to produce its beneficial effects on failing hearts by directly inhibiting cardiac NHE1 activity and thereby reducing intracellular [Na^+^].^9^^,^^10^ These inferences were based on measurements of pH_i_ and [Na^+^] not dissimilar to our experiments. For example, Baartscheer *et al.*^9^ found that EMPA, obtained from MCE, at 1 µM concentration inhibited NHE1 activity by 80%, based on a slowing of pH_i_ recovery after an ammonium pre-pulse. They also showed that EMPA caused a prompt ∼25% decrease in [Na^+^]_i_, ostensibly due to an inhibition of NHE1-dependent Na^+^ influx. These effects of EMPA were replicated in another study by the same group (Uthman *et al.*^10^), who additionally found that related SGLTi’s (DAPA and CANA) had similar effects on pH_i_ and [Na^+^]_i_. Independently, *in silico* mathematical simulations identified NHE1 as a target of EMPA’s cardiac actions, and indicated that the drug’s beneficial effects on the heart could be attributable to this mechanism.^38^ However, that study was not based on target identification experiments validating a direct molecular interaction between EMPA and NHE1; rather, it identified that an inhibition of NHE1 by EMPA would indeed be beneficial in heart failure, assuming that such interaction exists based on published data.

The reasons for the discrepancy between our data and previously published findings are unclear. Both studies used a similar fluorimetric approach on isolated cardiac ventricular myocytes to measure the recovery of pH_i_ from an acid-load imposed by an ammonium pre-pulse. One notable difference is that the previous study^9^ used a buffer formulation that has an inherently unstable pH, and therefore will drift over time. This instability is due to the inclusion of${\text{HCO}}_{3}^{-}$ (4.3 mM) in the absence of CO_2_, i.e. an out-of-equilibrium solution.^39^ In such a system, ${\text{HCO}}_{3}^{-}$ ions will attempt to reach equilibrium by protonating to form CO_2_ (and water), but any CO_2_ produced will escape, driving the system further towards CO_2_ production. This has the effect of gradually alkalinizing the solution. Since NHE1 is strongly dependent on extracellular pH,^40^ the measured NHE1 activity will not be consistent. To avoid this problem, we used a closed buffer system with no ${\text{HCO}}_{3}^{-}$ and no CO_2_, effectively clamping buffer pH to 7.4. Therefore, in a buffer which contains ${\text{HCO}}_{3}^{-}$ but not balanced by CO_2_, the time-dependence of solution pH will invariably introduce an error in measurements.

It is also plausible that different batches of EMPA may have varying degrees of purity, resulting in a lower concentration of bio-active compound in our experiments. To control for this, we obtained the drug from two independent sources: MCE (same manufacturer used in previous studies) and Boehringer. The purity and chemical structure of EMPA was verified using NMR, CD, and HPLC-MS, and confirmed to be identical for both sources, and of equally high purity (<1% impurities; Supplementary material online, *Figure S1*). We also confirmed that our source of EMPA, mEMPA, DAPA, and CANA were functionally active, as they inhibited glucose uptake in HEK293 cells overexpressing hSGLT2 (Supplementary material online, *Figure S2*). Our studies were also performed on a wider range of concentrations, and even at the highest dose of 30 µM (far in excess of the reported therapeutic range), EMPA had no meaningful inhibitory effect on NHE1.

Another possible reason for the discrepancy could relate to species differences in the drug’s target, NHE1. Differences in the primary sequence of other ion transporters are known to affect inhibitor sensitivity. For example, in the murine heart, two amino acid substitutions (at positions 111 and 122 in the α1 subunit) render NKA resistant to ouabain when compared to other mammals (i.e. rabbits, guinea pigs, humans).^41^ Uthman *et al.*^10^ suggest that the putative binding of EMPA to NHE1 is in the region of the Na^+^ binding site and, since this is highly conserved across mammalian species (>90% sequence homology), there is no evidence for a species-dependent difference in primary sequence that might alter EMPA affinity. In addition, there have been no reports in the literature of species-dependent differences in NHE1 inhibitor sensitivity.^42^^,^^43^ Finally, the previous studies of EMPA^9^^,^^10^ used the same concentration of the drug (1 µM) for measurements on rabbit, rat, and mouse hearts and myocytes, and produced consistent responses. In the present study, EMPA consistently had no actions on NHE1 in human cells (Supplementary material online, *Figure S4*), rat, mouse, and guinea pig hearts (*Figure 4A and B*), collectively arguing against a species-dependent difference.

Previous *in silico* modelling predicted high-affinity binding of EMPA to NHE1 involving the drug’s glucoside moiety occupying the Na^+^ binding site of NHE1.^10^ Such an interaction resembles that of acylguanidine-based NHE1 inhibitors, such as amiloride, cariporide, zoniporide, and others.^44^^,^^45^ The inhibitory potency of such drugs is related to (i) the ionization state of the acylguanidine group, which is cationic at acidic pH and (ii) structure, which resembles that of a tri-hydrated Na^+^ ion.^29^^,^^44^ A new class of non-acylguanidine inhibitors has been recently described (SL591227^46^ and 9t^47^) that is predicted to bind NHE1 via their imidazole motif, a surrogate for the acylguanidine group.^46^^,^^48^ Since EMPA does not possess either of these motifs, an interaction with NHE1 would be surprising and certainly not canonical.

EMPA has been shown to also inhibit SGLT1 with an IC_50_ of 8.3 µmol/L.^1^ Given that SGLT1 is expressed in the heart,^47^ it is plausible that inhibition of SGLT1 may have affected our results. However, evidence for the expression of this transporter specifically in the cardiac myocyte in healthy hearts is lacking, as localization studies place it in cardiac capillaries rather than cardiomyocytes.^49^ In addition, of the four concentrations of EMPA tested in this study, two of these (1 and 3 µM) were well below the IC_50_ of SGLT1. The effect of EMPA at these lower concentrations on NHE1 flux is comparable to the effects observed at 10 and 30 µM EMPA, concentrations at which SGLT1 may be inhibited. This suggests that the lack of NHE1 inhibition by EMPA is independent of a possible SGLT1 inhibition (if it is indeed expressed) in cardiac myocytes.

Evidence for the beneficial effects of EMPA in the heart are numerous, both in humans^2^^,^^20^^,^^23^ and in animals^3–5^. A number of alternative mechanisms have been postulated to explain the beneficial effects of SGLT2i’s in heart failure. These include changes in metabolic substrate preference and more efficient mitochondrial energy production,^50^^,^^51^ and improved ventricular loading secondary to enhanced renal function.^52^^,^^53^ Recently, it was also shown that EMPA can increase nitric oxide (NO) signalling in the heart.^54^ However, based on the results of this study, the observed cardioprotective effects cannot be explained by a direct inhibitory interaction of SGLT2 inhibitors, especially EMPA, with cardiac NHE1 and subsequent lowering of intracellular [Na^+^].

## Supplementary material

Supplementary material is available at *Cardiovascular Research* online.

## Supplementary Material

cvaa323_Supplementary_Figures

## Notes

Translational perspective Heart failure remains a huge clinical burden. Clinical trials of sodium-glucose co-transporter 2 (SGLT2) inhibitors in patients with diabetes and heart failure have reported highly significant cardiovascular benefit that appears independent of improved glycaemic control. As SGLT2 is not expressed in the heart, the mechanism by which SGLT2 inhibitors are cardioprotective remains unknown. Understanding this mechanism is clearly essential as the use of SGLT2 inhibitors in non-diabetics is increasing and a better understanding may allow refinement of therapeutic approaches in both heart failure with preserved ejection fraction (HFpEF) and heart failure with reduced ejection fraction (HFrEF). One suggested mechanism that has received significant attention, inhibition of cardiac Na^+^/H^+^ exchanger, is investigated here.
