# Supplementary material for: Off-target effects of sodium-glucose co-transporter 2 blockers: empagliflozin does not inhibit Na+/H+ exchanger-1 or lower [Na+]i in the heart
Source: Cardiovasc Res. 2020 Nov 2;117(14):2794–806. doi: 10.1093/cvr/cvaa323 (PMC8683707; doi:10.1093/cvr/cvaa323)
Supplement: cvaa323_Supplementary_Figures [file cvaa323_supplementary_figures.pdf]

SUPPLEMENTARY FIGURES

Off-target effects of SGLT2 blockers: empagliflozin does not inhibit Na^+/H^+ exchanger-1 or lower $[Na^+]_i$ in the heart

Short title: EMPA does not inhibit cardiac NHE1 or lower $[Na^+]_i$

Yu Jin CHUNG[1], Kyung Chan PARK[2], Sergiy TOKAR[1], Thomas R. EYKYN[1,3], William FULLER[4], Davor PAVLOVIC[5], Pawel SWIETACH[2], Michael J. SHATTOCK[1*]

(1) British Heart Foundation Centre of Research Excellence, King's College London, United Kingdom

(2) Burdon Sanderson Cardiac Science Centre, Department of Physiology, Anatomy and Genetics, University of Oxford, United Kingdom

(3) School of Biomedical Engineering and Imaging Sciences, King's College London, United Kingdom

(4) Institute of Cardiovascular & Medical Sciences, University of Glasgow, United Kingdom

(5) Institute for Cardiovascular Sciences, University of Birmingham, United Kingdom

*Address for correspondence: The Rayne Institute, 4th Floor, Lambeth Wing, St Thomas' Hospital, London SE1 7EH, UK. Email: michael.shattock@kcl.ac.uk. Telephone: +44 (0)20 7188 0945

Manuscript category: Original Article

Word Count: 8534

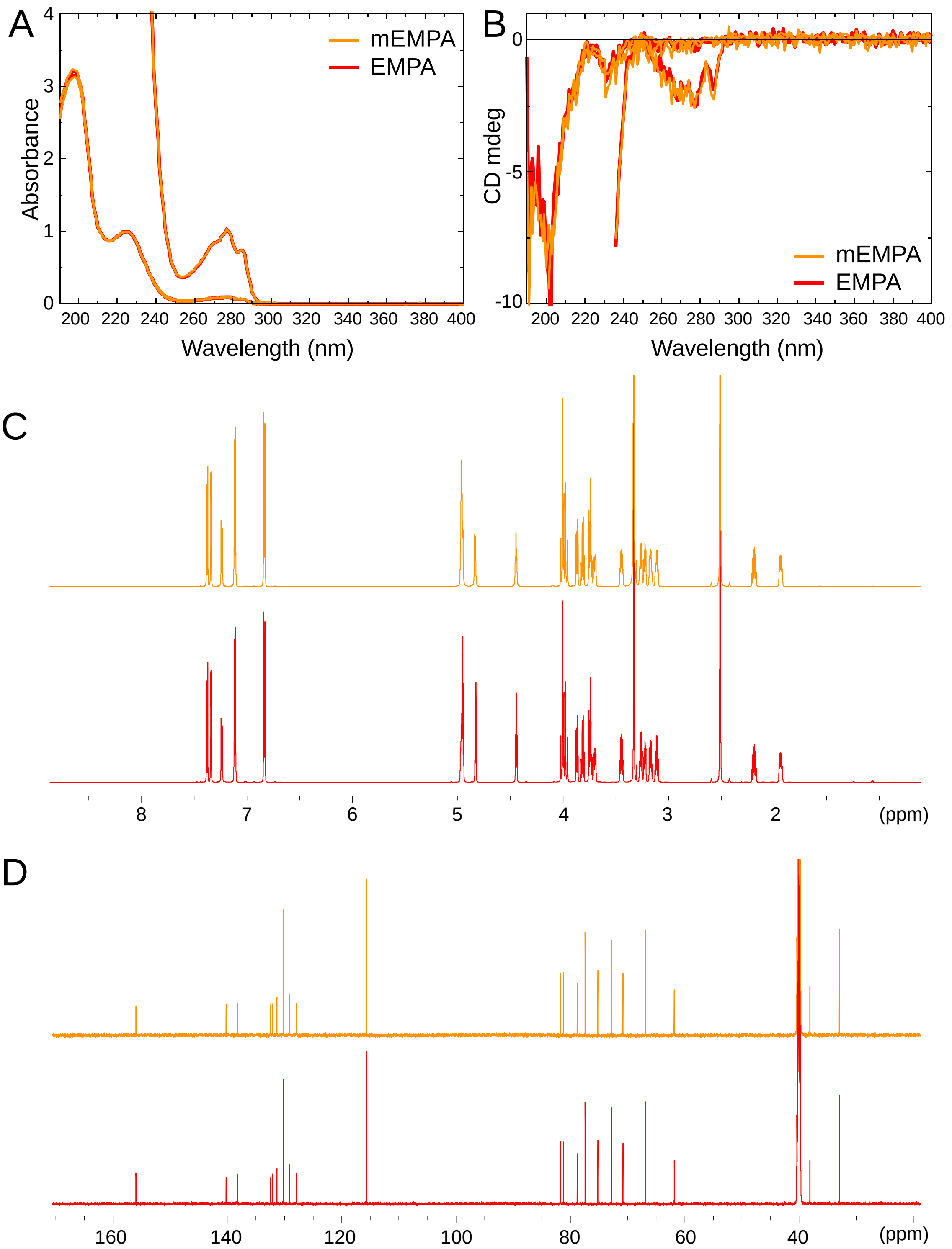

Figure S1. (A) UV absorbance and **(B)** Circular Dichroism (CD) of mEMPA (yellow) and EMPA (red) dissolved in methanol. Spectra were acquired at 23°C on the Applied Photophysics Chirascan Plus spectrometer (Leatherhead, UK) using a 0.5 mm (Starna Scientific Ltd) Quartz Suprasil rectangular cuvette in the region 400-180nm. UV & CD spectra were solvent baseline corrected. **(C)** ^{1}H NMR spectra and **(D)** ^{13}C spectra of mEMPA (yellow) and EMPA (red) dissolved in DMSO-d6 acquired on a Bruker 800MHz spectrometer.

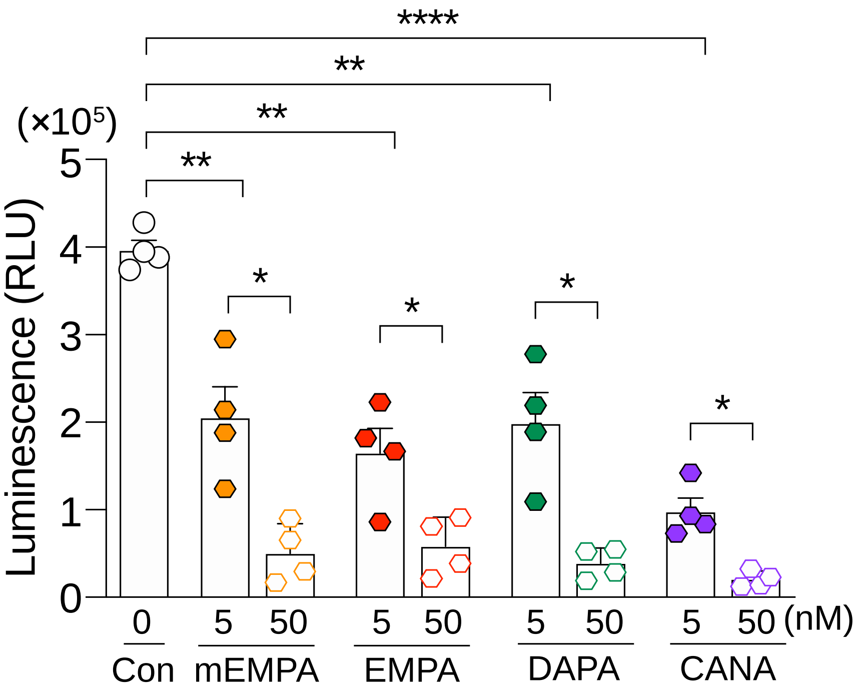

Figure S2. SGLT2 inhibitors inhibit glucose uptake. Dose dependent inhibition of glucose uptake in HEK293 cells over expressing human SGLT2 when treated with increasing dose of SGLT2 inhibitors mEMPA, EMPA, DAPA and CANA. Con = control. n = 4 replicates per condition. * $p<0.05$, ** $p<0.01$ by two-tail, unpaired student's *t*-test.

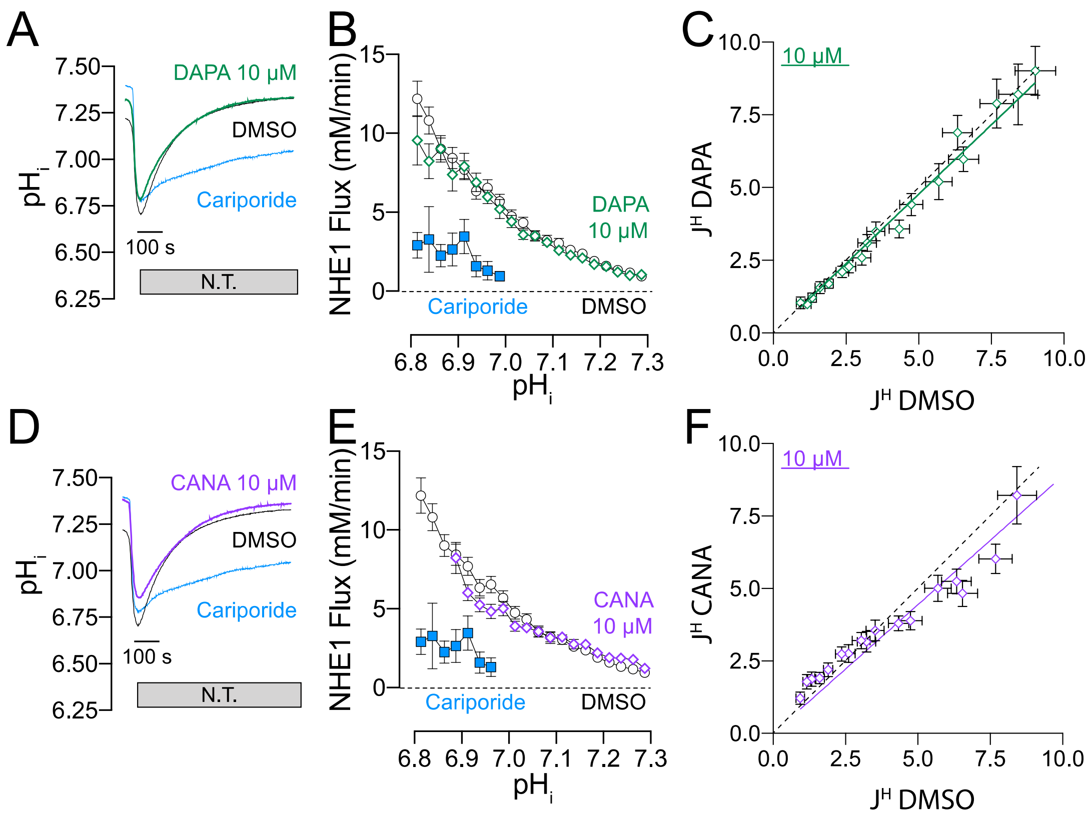

Figure S3. SGLT2 inhibitors do not inhibit NHE1 flux. pH_i recovery time course **(A)** and **(D)** and NHE1 flux as a function of pH_i **(B)** and **(E)** measured in rat cells exposed to acute NH_4^+ acid load and wash-out and pre-treated with DMSO, 10 µM cariporide or 10 µM DAPA or CANA. **(C)** and **(F)** Acid extrusion flux (J^H; mM/min) plotted at matching pH_i for control (DMSO) and in presence of 10 µM DAPA or CANA. Solid line through data shows best fit through origin, and the slope describes NHE1 activity in the presence of drug (where 1.0 is no inhibition, indicated in broken black line). n = 31-43 cells from 2 rat per condition (Cariporide n = 6 cells from 1 rat). N.T. = Normal Tyrode's buffer. All data mean ± SEM (error bar not included in *(A)* and *(D)* for clarity).

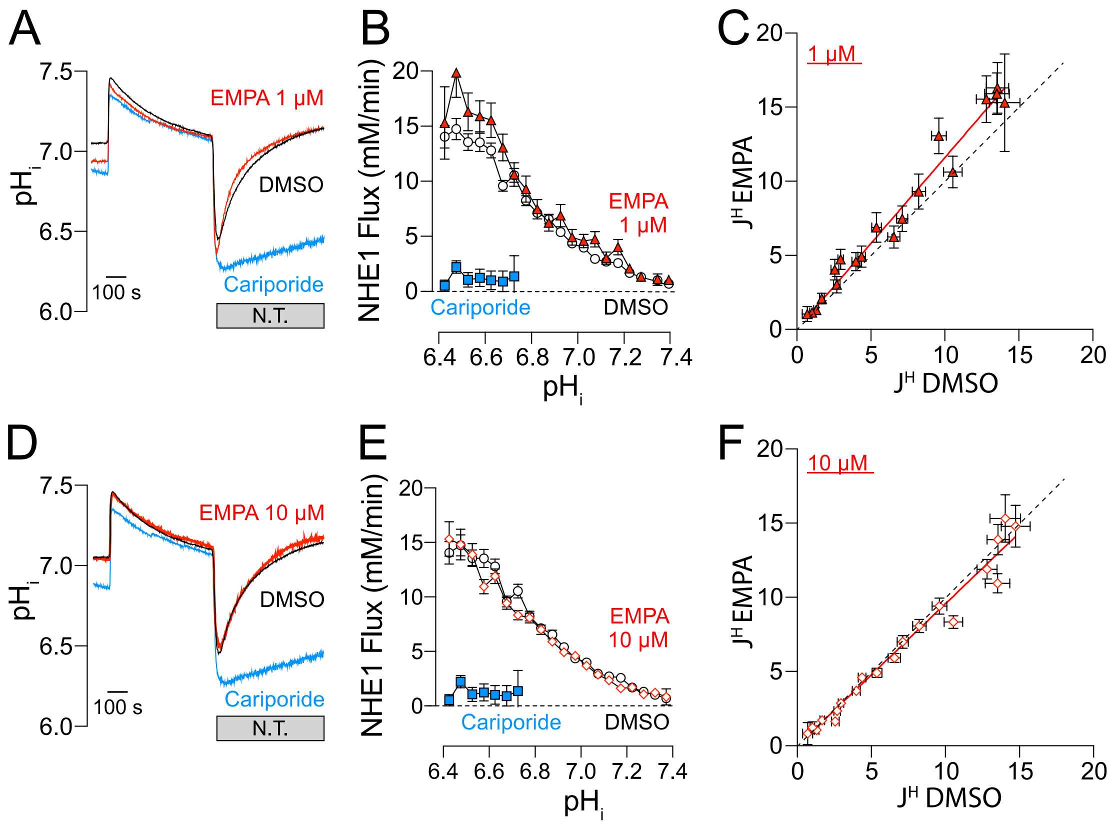

Figure S4. EMPA does not inhibit human NHE1 flux in HCT116 cells. pH_i recovery time course **(A)** and **(D)** and NHE1 flux as a function of pH_i **(B)** and **(E)** measured in human HCT116 cells exposed to acute NH_4^+ acid load and wash-out and pre-treated with DMSO, 30 µM cariporide or 1 or 10 µM EMPA. **(C)** and **(F)** Acid extrusion flux (J^H; mM/min) plotted at matching pH_i for control (DMSO) and in presence of 1 or 10 µM EMPA. Solid red line through data shows best fit through origin, and the slope describes NHE1 activity in the presence of drug (where 1.0 is no inhibition, indicated in broken black line). N.T. = Normal Tyrode's buffer. n = 305 (control), 75 (1 µM EMPA), 245 (10 µM EMPA) and 45 (cariporide) cells. All data mean ± SEM (error bar not included in *(A)* and *(D)* for clarity).

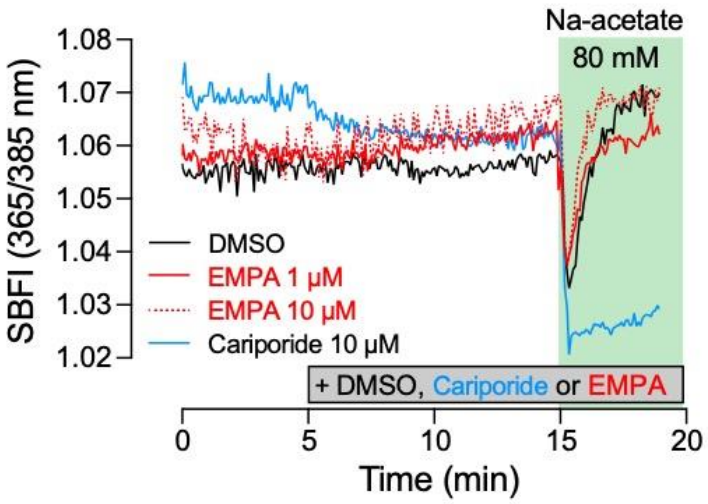

Figure S5. Unaltered time course shown in Fig 3B. For calculating the pH_i-correction of SBFI ratio, we assumed that the initial pH_i drop attained with 80 mM acetate was the same in the presence of EMPA, cariporide and in drug free conditions. Since cariporide blocks the NHE1-dependent Na entry evoked by acidosis, the ratio immediately before and after the switch to acetate was assumed to correpond to the same level of $[Na^+]_i$. Thus it was possible to scale the ratio in the presence of acetate (shaded area) for all four experiumental groups.
